# A Personalized Mobile Health Program for Type 2 Diabetes During the COVID-19 Pandemic: Single-Group Pre–Post Study

**DOI:** 10.2196/25820

**Published:** 2021-07-09

**Authors:** Ian Yi Han Ang, Kyle Xin Quan Tan, Clive Tan, Chiew Hoon Tan, James Wei Ming Kwek, Joanne Tay, Sue Anne Toh

**Affiliations:** 1 Saw Swee Hock School of Public Health National University of Singapore and National University Health System Singapore Singapore; 2 NOVI Health Singapore Singapore; 3 Singapore Armed Forces Singapore Singapore; 4 Yong Loo Lin School of Medicine National University of Singapore and National University Health System Singapore Singapore; 5 Regional Health System Office National University Health System Singapore Singapore

**Keywords:** type 2 diabetes, prediabetic state, text messaging, mobile applications, glycated hemoglobin A, HbA1c, blood glucose, body mass index, mHealth, COVID-19, diabetes, intervention, self-management, chronic disease, outcome

## Abstract

**Background:**

With increasing type 2 diabetes prevalence, there is a need for effective programs that support diabetes management and improve type 2 diabetes outcomes. Mobile health (mHealth) interventions have shown promising results. With advances in wearable sensors and improved integration, mHealth programs could become more accessible and personalized.

**Objective:**

The study aimed to evaluate the feasibility, acceptability, and effectiveness of a personalized mHealth-anchored intervention program in improving glycemic control and enhancing care experience in diabetes management. The program was coincidentally implemented during the national-level lockdown for COVID-19 in Singapore, allowing for a timely study of the use of mHealth for chronic disease management.

**Methods:**

Patients with type 2 diabetes or prediabetes were enrolled from the Singapore Armed Forces and offered a 3-month intervention program in addition to the usual care they received. The program was standardized to include (1) in-person initial consultation with a clinical dietitian; (2) in-person review with a diabetes specialist doctor; (3) 1 continuous glucose monitoring device; (4) access to the mobile app for dietary intake and physical activity tracking, and communication via messaging with the dietitian and doctor; and (5) context-sensitive digital health coaching over the mobile app. Medical support was rendered to the patients on an as-needed basis when they required advice on adjustment of medications. Measurements of weight, height, and glycated hemoglobin A_1c_ (HbA_1c_) were conducted at 2 in-person visits at the start and end of the program. At the end of the program, patients were asked to complete a short acceptability feedback survey to understand the motivation for joining the program, their satisfaction, and suggestions for improvement.

**Results:**

Over a 4-week recruitment period, 130 individuals were screened, the enrollment target of 30 patients was met, and 21 patients completed the program and were included in the final analyses; 9 patients were lost to follow-up (full data were not available for the final analyses). There were no differences in the baseline characteristics between patients who were included and excluded from the final analyses (age category: *P*=.23; gender: *P*=.21; ethnicity: *P*>.99; diabetes status category: *P*=.52, medication adjustment category: *P*=.65; HbA_1c_ category: *P*=.69; BMI: *P*>.99). The 21 patients who completed the study rated a mean of 9.0 out of 10 on the Likert scale for both satisfaction questions. For the Yes-No question on benefit of the program, all of the patients selected “Yes.” Mean HbA_1c_ decreased from 7.6% to 7.0% (*P*=.004). There were no severe hypoglycemia events (glucose level <3.0 mmol/L) reported. Mean weight decreased from 76.8 kg to 73.9 kg (*P*<.001), a mean decrease of 3.5% from baseline weight. Mean BMI decreased from 27.8 kg/m^2^ to 26.7 kg/m^2^ (*P*<.001).

**Conclusions:**

The personalized mHealth program was feasible, acceptable, and produced significant reductions in HbA_1c_ (*P*=.004) and body weight (*P*<.001) in individuals with type 2 diabetes. Such mHealth programs could overcome challenges posed to chronic disease management by COVID-19, including disruptions to in-person health care access.

## Introduction

Close to half a billion people in the world live with type 2 diabetes, and this prevalence is expected to increase by 25% by 2030 [1]. An estimated 430,000 (14.4% prevalence) Singapore residents aged 21 years and older had type 2 diabetes in 2015, and it has been estimated that the number will grow to 820,000 in the year 2035 (22.7% prevalence), assuming no change to the current landscape [2]. In addition, an estimated 560,000 (18.6% prevalence) Singapore residents in 2015 have prediabetes, of whom an estimated 490,000 (16.2% prevalence) were undetected. Type 2 diabetes has been identified as a chronic disease whose patients persistently incur high health care costs [3]. Effective scalable prevention measures are thus urgently needed to prevent and better manage type 2 diabetes to reduce its burden.

Lifestyle and behavior modification interventions for the prevention and management of type 2 diabetes have been shown to be effective in reducing risk of disease progression [4,5]. Executed well, lifestyle and behavior modification intervention programs can even have long-term sustained beneficial effects in decreased diabetes incidence and associated complications [6]. Such programs have traditionally been structured with a high frequency of in-person group-based sessions over a long duration (at least 6 months) [4,5]. Such programs can require a sizeable multidisciplinary professional team to run, which is costly [7,8], thereby limiting the scalability and sustainability of such interventions.

Simultaneously, many of these traditional lifestyle and behavior modification intervention programs have been found to have low participation rates [4] and high attrition [9]. Reasons for dropping out from such intervention programs include conflict between work schedules and center's hours of operation, distance to center, forgetfulness, lack of familiarity with the center and services, and apathy toward diabetes education [10]. Potential solutions to these barriers include running the program in the community [11,12], with reduced intensity [13], and leveraging mobile health (mHealth) interventions [14].

The use of mHealth for lifestyle and behavior modification interventions capitalizes on easily rolled out technologies to make communication and self-management education components easily accessible and independent of location. Over the years, mHealth interventions have progressed from using phone calls, text messages, and internet websites to, more recently, smartphone apps. The use of mHealth interventions for chronic disease care and management has been well-received with high acceptability and engagement [15-17]. In the care and management of type 2 diabetes, mHealth interventions have been successful in achieving improvements in clinical outcomes [18,19]. The use of adaptable feedback on behaviors with tailored messaging in mHealth interventions further allows for personalization according to the needs and preferences of patients [20,21]. Such a patient-centered approach of mHealth interventions could improve motivation in patients to make lifestyle and behavioral modifications and to sustain the changes made [22,23].

The use of wearable sensors in mHealth interventions provides real-time tracking and monitoring in patients with type 2 diabetes. Self-monitoring of blood glucose level, either by finger-stick or continuous glucose monitoring (CGM) technology, has been shown to be useful in helping patients improve their diabetes control [24-27]. Blood glucose data logged in mHealth apps can be consolidated with app-recorded diet and physical activity data and have been found to help facilitate self-care in patients at risk of or with type 2 diabetes [28,29]. Drawing on advancements in technology, integration of various successful features could bring about synergistic improvements in mHealth interventions for the management of type 2 diabetes.

Given the increasing burden and cost of uncontrolled type 2 diabetes and related complications, there is a great urgency for scalable and effective solutions that reduce such a burden and cost [30,31]. In response to this need, a personalized mHealth-anchored intervention program was designed and implemented in patients with type 2 diabetes or prediabetes. This study aimed to evaluate the effectiveness and feasibility of this personalized mHealth program in improving glycemic control and enhancing care experience in diabetes management.

## Methods

### Site and Population

The program was conducted in Singapore, a city-state in tropical Southeast Asia with a population of 5.64 million people [32]. The patients were recruited from the Singapore Armed Forces in collaboration with their Headquarters Medical Corps. The Singapore Armed Forces provides primary health care services within military camps for its full-time service personnel and conscripts and a range of risk-based health screening programs for personnel in older age ranges. These older personnel with chronic health conditions are also free to obtain care from the national health care system outside of the Singapore Armed Forces.

The patients were recruited from active full-time service personnel and conscripts. Invitation to participate in the program was conducted by the Singapore Armed Forces’ Headquarters Medical Corps through a series of intranet publicity advertisements posted over 4 weeks in February 2020. Interested patients were screened by the Headquarters Medical Corps and were enrolled into the program if they were interested and met the eligibility criteria (of having type 2 diabetes or prediabetes). The patients were deemed to have (1) type 2 diabetes, if they had glycated hemoglobin (HbA_1c_) ≥6.5% in the past 1 year or if they were on medication for type 2 diabetes, or (2) prediabetes, if they had an HbA_1c_ level in the range 5.7% to 6.4% in the past year, and they were not taking any medications for type 2 diabetes. The enrollment target was set at 30 individuals, which was deemed to be sufficient to assess the feasibility and acceptability of such an intervention in a pilot program [33,34].

### Intervention Program

The Singapore Armed Forces’ Headquarters Medical Corps worked with NOVI Health, a health care technology start-up based in Singapore, to provide their proprietary mHealth program to the enrolled population.

**Figure 1:**
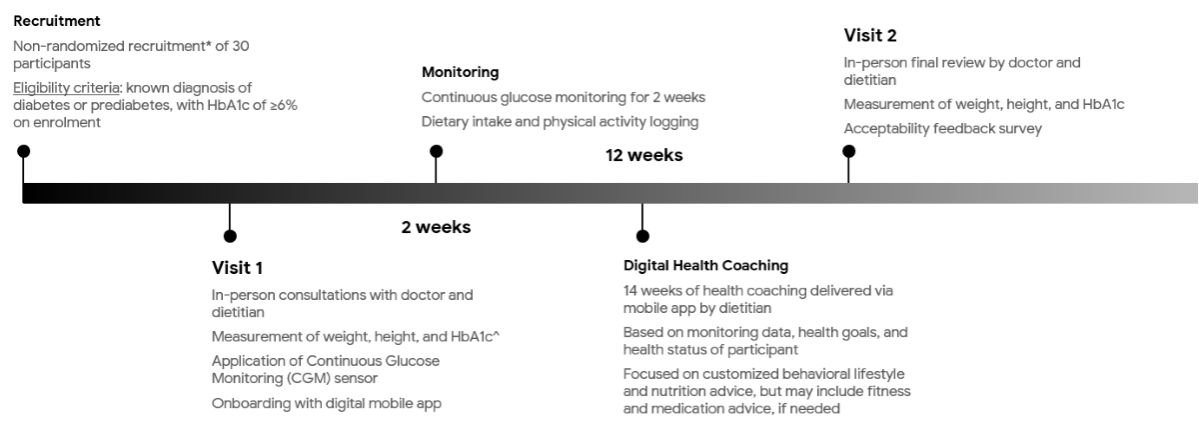
Intervention program timeline and protocol. *Recruitment with a series of publicity advertisements on the Singapore Armed Forces’ intranet. ^Glycated hemoglobin level (HbA_1c_) was measured if there was no valid reading within the prior 3 months.

**Figure 2:**
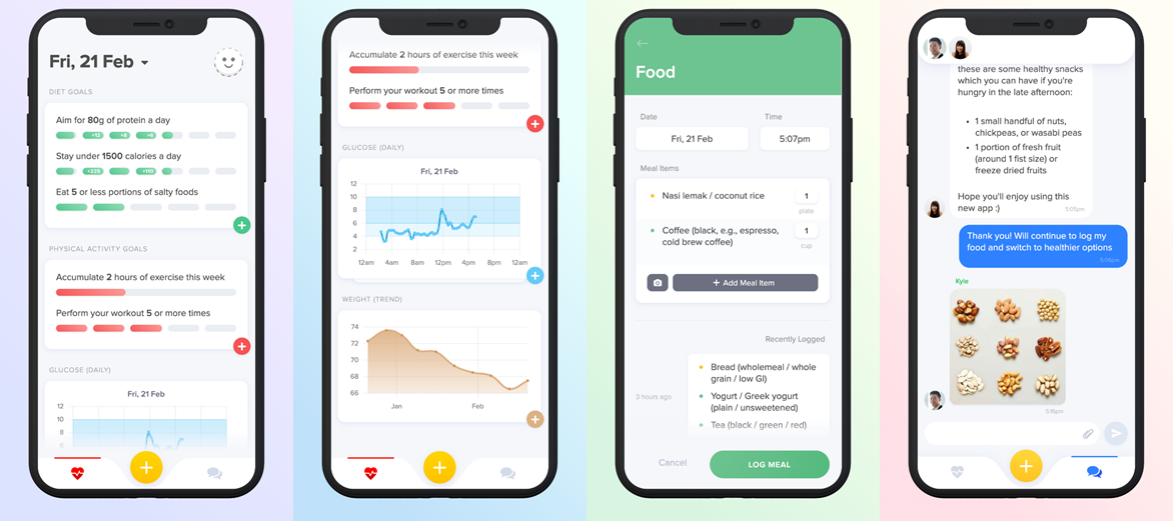
Screenshots of mobile app dashboard with diet and physical activity goals, real-time continuous glucose monitoring data, dietary intake and physical activity logs, and personalized recommendations delivered through the messaging function in the mobile app.

All eligible patients were offered the 3-month intervention program in addition to the usual care that they received for their type 2 diabetes or prediabetes (Figure 1). The program was standardized to include the following components: (1) in-person initial consultation with a clinical dietitian that served as a health coach, (2) in-person review with a diabetes specialist doctor, (3) 1 Abbott Freestyle Libre CGM device that provided monitoring in the first 2 weeks, (4) access to the mobile app that allowed dietary intake and physical activity tracking and communication via text messaging with the dietitian and doctor, and (5) context-sensitive digital health coaching provided by the dietitian over the mobile app (Figure 2).

In the first in-person visit to the clinic (Visit 1), the patients had a consultation with the dietitian to set their health goals and discuss behavioral lifestyle changes that could be made. The patients also had a consultation with the diabetes specialist doctor, which allowed for the review of comorbidities and medication regime. The patients were also provided with 1 CGM device and were guided on how to use the device for glucose monitoring and how to provide the care team with access to their real-time CGM data.

In the subsequent 3 months after Visit 1, the patients were free-living and used the mobile app to log their dietary intake and physical activity. The dietitian and doctor were able to view the CGM, dietary intake, and physical activity data together with available information on the patients’ health status (information on HbA_1c_, comorbidities, and medication regime). This allowed them to deliver timely personalized recommendations through the messaging function of the mobile app. The health coaching via the mobile app was led by the clinical dietitian, with input from the fitness coaches provided when needed, and with medical oversight from the reviewing diabetes specialist doctor. Medical support via the mobile app was also provided to the patients on an as-needed basis (if they experienced hypoglycemia requiring medication adjustments or if they required advice on the adjustment of medications, etc). After 3 months, the patients returned to the clinic for their second in-person visit (Visit 2) to meet with the diabetes specialist doctor and dietitian for final review.

Patient recruitment for the program started in February 2020 and the program ran from March 2020 to June 2020. The recruitment period and the first month of the intervention program corresponded with a worsening COVID-19 situation in Singapore through the months of February and March 2020. This culminated in a national-level lockdown, which started on April 7, 2020 and ended on June 1, 2020, coinciding with the second and third months of the intervention program. The 2 in-person visits to the clinic (Visits 1 and 2) happened to have been scheduled in the periods before and after the lockdown in Singapore and were, therefore, not impacted.

### Outcome Measurements

Measurements of weight, height, and HbA_1c_ were conducted at Visits 1 and 2. Weight and height were measured by a trained nurse, using a Surgico Healthweigh machine. At the end of the program at Visit 2, the patients were asked to complete a short acceptability feedback survey to understand the motivation for joining the program, their satisfaction, and suggestions for improvement. The question on motivation, “What was your primary motivation for signing up for this program?” had 4 options: “1. Wanted to get my diabetes under control, 2. Wanted to get dietary advice for my diabetes, 3. Wanted to lose weight, 4. Was asked to participate by HQMC.” There were 2 satisfaction questions rated on a 10-point Likert scale, “How satisfied were you with the program?” and “How likely are you to recommend this program to your colleague?” A fourth question “Do you think that other servicemen would benefit from this program?” was a Yes-No question asking the patients on whether the program would be beneficial to others.

### Data Analysis

Patients were considered to have completed the study and included in the final outcomes analyses if they had completed the full 14 weeks of the intervention program, with weight, height, and HbA_1c_ measurements at baseline (Visit 1) and at completion of the program (Visit 2). Means were calculated for continuous baseline characteristic variables. Due to nonnormality of data, the Wilcoxon signed rank test was used to compare means between those who completed the study and those that were lost to follow-up and excluded. Proportions were calculated for baseline characteristics that were categorical variables. The Fisher exact test was used if counts were less than 5 to compare distributions of those who completed the study and those who were lost to follow-up and excluded.

The final analyses of main outcomes of interest for acceptability and effectiveness were limited to only the those who completed the study. For the outcomes of HbA_1c_, weight, and BMI, the patients were further split for subgroup analyses: (1) by baseline HbA_1c_ ≤7% or >7%, and (2) by baseline BMI<27.5 kg/m^2^ (normal and overweight) or ≥27.5 kg/m^2^ (obese). The HbA_1c_ cut-off was selected based on the HbA_1c_ threshold set by major clinical guidelines for what is considered good diabetes control [35-37], and the BMI threshold was selected based on what is considered obese in the Asian population [38]. Due to nonnormality of data, the Wilcoxon signed rank test was used for the paired comparisons of the main outcomes at Visit 1 and Visit 2, with significance level set at α=.05. Analyses were conducted in R (version 3.6.1). Means and standard deviations of the main outcomes are presented, along with *P* values, where applicable. Calculation of type II beta errors were also conducted for the main outcomes of interest using G*Power (version 3.1.9.4), for a 2-tailed test according to a Laplace parent distribution and α=.05. Beta errors >.2 are indicated.

### Ethics Approval

This study was conducted as part of a program evaluation. The data collected were presented at the Singapore Armed Forces’ Joint Medical Committee for Research and approved for exemption from full review at the Institutional Review Board.

## Results

### Baseline Characteristics

Over the 4-weeks recruitment period in February 2020, there were 130 individuals screened, of whom 30 met eligibility criteria and were interested in participating in the program. Of the 30 enrolled patients, 7 were lost to follow-up, and 2 had completed the program but did not have complete measurements from Visit 2. As such, 21 patients were included in the final outcome analyses. There were no significant differences in any of the baseline characteristics (age category: *P*=.23; gender: *P*=.21; ethnicity: *P*>.99; diabetes status category: *P*=.52, medication adjustment category: *P*=.65; HbA_1c_ category: *P*=.69; BMI: *P*>.99) between the patients who were excluded and those who were included in the final outcome analyses (Table 1). The majority of the patients were male. The majority of the patients had diabetes; the patients who had diabetes were all on glucose lowering medication upon enrollment into the program. While there were patients who were on insulin therapy upon entry into the program, no patient started insulin during the program. There were 5 patients who had their medication adjusted. One patient’s insulin dosage was reduced, 2 patients had their medication (sulfonylurea) switched to another oral antihyperglycemic medication to reduce hypoglycemic risk, and 2 patients had 1 oral antihyperglycemic medication added to their existing regime. Approximately two-thirds of the patients had baseline HbA_1c_ >7%, and approximately half had BMIs that placed them in the obese category.

**Table 1:** Baseline characteristics of all enrolled patients, patients lost to follow-up, and patients who completed the program and were included in the final analyses.

Characteristics		All recruited (n=30)	Lost to follow-up (n=9)	Completed (n=21)	*P* value
Age (years), mean (range)		49.1 (21-64)	43.7 (21-61)	51.4 (32-64)	.054
**Age, n (%)**					.23
	<50 years	17 (57)	7 (78)	10 (48)	
	≥50 years	13 (43)	2 (22)	11 (52)	
**Gender, n (%)**					.21
	Female	9 (30)	1 (11)	8 (38)	
	Male	21 (70)	8 (89)	13 (62)	
**Ethnicity, n (%)**					>.99
	Chinese	24 (80)	8 (89)	16 (76)	
	Malay	1 (3)	0 (0)	1 (5)	
	Indian/Pakistani	5 (17)	1 (11)	4 (19.0)	
**Diabetes status, n (%)**					.52
	Prediabetes	2 (7)	1 (11)	1 (5)	
	Type 2 diabetes	28 (93)	8 (89)	20 (95)	
**Medication adjustments, n (%)**					.65
	Adjusted	8 (27)	3 (33)	5 (24)	
	Not adjusted	22 (73)	6 (67)	16 (76)	
Baseline HbA_1c_^a^ (%), mean		7.7	7.9	7.6	>.99
**Baseline HbA_1c_ category, n (%)**					.69
	≤7%	11 (37)	4 (44)	7 (33)	
	>7%	19 (63)	5 (56)	14 (67)	
Baseline BMI^b^ (kg/m^2^), mean		27.9	28.2	27.8	.96
**Baseline BMI category, n (%)**					>.99
	<27.5 kg/m^2^	15 (50)	4 (44)	11 (52)	
	≥27.5 kg/m^2^	15 (50)	5 (56)	10 (48)	
Baseline weight (kg), mean		77.7	79.7	76.8	.82

^a^HbA_1c_: glycated hemoglobin.

^b^BMI: body mass index.

### Acceptability Feedback

For the multiple-choice question on motivation, 48% of patients (10/21) selected “Wanted to get my diabetes under control,” 19% of patients (4/21) selected “Wanted to get dietary advice for my diabetes,” 3 (14% of patients (3/21) patients selected “Wanted to lose weight,” and 19% of patients (4/21) selected “Was asked to participate by HQMC.” The patients rated a mean of 9.0 out of 10 on the Likert scale for both satisfaction questions. For the Yes-No question on the benefit of the program, all patients selected “Yes.”

### Effectiveness Outcomes

For all 21 who completed the study, mean HbA_1c_ decreased from 7.6% to 7.0% (*P*=.004) (Table 2). Mean weight had decreased from 76.8 kg to 73.9 kg (*P*<.001), which was a mean decrease of 3.5% (SD 3.2%) from baseline. Mean BMI had decreased from 27.8 kg/m^2^ to 26.7 kg/m^2^ (*P*<.001).

**Table 2:** Comparison of HbA_1c_, weight, and BMI at Visit 1 and Visit 2 for all patients who completed the study.

Outcome	Visit 1, mean (SD)	Visit 2, mean (SD)	*P* value
HbA_1c_^a^ (%)	7.6 (1.1)	7.0 (0.8)	.004
Weight (kg)	76.8 (15.6)	73.9 (13.8)	<.001
BMI^b^ (kg/m^2^)	27.8 (5.4)	26.7 (4.8)	<.001

^a^HbA_1c_: glycated hemoglobin.

^b^BMI: body mass index.

### Subgroup Analyses by Baseline HbA_1c_ Category

For patients who had baseline HbA_1c_ ≤7%, there was no statistically significant change in HbA_1c_ upon completion of the 3-month intervention program (*P*=.67), but the beta error was found to be >.2 (Table 3). However, mean weight decreased from 75.0 kg to 73.0 kg (*P*=.02; mean decrease 3.9%, SD 3.7%). Mean BMI decreased from 26.8 kg/m^2^ to 26.1 kg/m^2^ (*P*=.02). For the patients who had baseline HbA_1c_ >7%, mean HbA_1c_ decreased from 8.1% to 7.2% (*P*=.005). Mean weight also decreased from 77.8 kg to 74.3 kg (*P*=.006), which was a mean decrease of 2.5% (SD 1.8%) from the baseline weight. Mean BMI decreased from 28.3 kg/m^2^ to 27.1 kg/m^2^ (*P*=.006).

**Table 3:** Comparison of Visit 1 and Visit 2 characteristics for patients who had baseline HbA_1c_ ≤7% or >7%.

Outcome	Baseline HbA_1c_ ≤7% (n=7)				Baseline HbA_1c_ >7% (n=14)		
	Visit 1, mean (SD)	Visit 2, mean (SD)	*P* value	Visit 1, mean (SD)		Visit 2, mean (SD)	*P* value
HbA_1c_^a^ (%)	6.7 (0.3)	6.6 (0.6)	.67^b^	8.1 (1.0)		7.2 (0.8)	.005
Weight (kg)	75.0 (13.5)	73.0 (12.2)	.02	77.8 (16.9)		74.3 (14.9)	.006
BMI^c^ (kg/m^2^)	26.8 (5.1)	26.1 (4.8)	.02	28.3 (5.7)		27.1 (4.9)	.006

^a^HbA_1c_: glycated hemoglobin.

^b^Type II beta error >.2.

^c^BMI: body mass index.

### Subgroup Analyses by Baseline BMI Category

There were no statistically significant changes in HbA_1c_, weight, or BMI for patients who were in the normal and overweight BMI category, after the intervention in Visit 2, but beta errors were found to be >.2 (Table 4). For the patients who were in the obese BMI category, mean HbA_1c_ decreased from 7.6% to 6.8% (*P=*.006). Mean weight also decreased from 89.3 kg to 84.1 kg (*P=*.002, mean decrease 5.9%, SD 2.2%). Mean BMI decreased from 32.5 kg/m^2^ to 30.6 kg/m^2^ (*P=*.002).

**Table 4:** Comparison of Visit 1 and Visit 2 characteristics for patients who had baseline BMI <27.5 kg/m^2^ (normal and overweight) or ≥27.5 kg/m^2^ (obese).

Outcome	Normal and overweight (n=11)				Obese (n=10)		
	Visit 1, mean (SD)	Visit 2, mean (SD)	*P* value	Visit 1, mean (SD)		Visit 2, mean (SD)	*P* value
HbA_1c_^a^ (%)	7.7 (1.3)	7.3 (0.9)	.14^b^	7.6 (0.9)		6.8 (0.6)	.006
Weight (kg)	65.5 (8.7)	64.6 (8.1)	.07^b^	89.3 (11.1)		84.1 (11.2)	.002
BMI^c^ (kg/m^2^)	23.5 (2.1)	23.2 (2.03)	.07^b^	32.5 (3.6)		30.6 (3.7)	.002

^a^HbA_1c_: glycated hemoglobin.

^b^Type II beta error >.2.

^c^BMI: body mass index.

### Complications

During the study, the patients had no hospitalization episodes for any diabetes-related complications. There were no severe hypoglycemia (glucose level <3 mmol/L) events observed or reported.

## Discussion

### General

This study evaluated a real-world personalized mHealth-anchored intervention program for feasibility, acceptability, and effectiveness for diabetes management. The program garnered a lot of interest and the enrollment target was met fairly quickly (in less than a month). The program was implemented as planned in spite of the disruptions from COVID-19. The program also received high patient ratings of satisfaction and perceived benefit from participation in the program. The patients achieved a significant reduction in HbA_1c_ in 3 months, ending the program with an average HbA_1c_ of 7%. Reduction of HbA_1c_ levels to ≤7% is consistent with the glycemic target set by most clinical guidelines [35,36] and has been shown to reduce microvascular [39-43] and macrovascular [44] complications in individuals with type 2 diabetes. Patients in the study also achieved weight loss over 3 months that met the clinically significant threshold of 3% [45,46]. Such reductions have been observed to lead to improvements in cardiovascular risk factors such as glycemic control, systolic and diastolic blood pressure, as well as with respect to low-density lipoprotein and high-density lipoprotein cholesterol levels [47].

The improvements observed were achieved in patients who had known type 2 diabetes and prediabetes and who were receiving usual care and on existing medications. This suggests that there could be a role for a personalized mHealth program for patients with diabetes, even those receiving usual medical care for their diabetes. Such a program could improve control of diabetes and further reduce the risk of microvascular and macrovascular complications. In the subgroups of patients with glycemic and BMI measures above the ideal range, the impact of the personalized mHealth program was even greater. These results were not unexpected as the patients with starting HbA_1c_ and weight values that were further from target were likely to have more room for improvement. The mHealth program could benefit most individuals with diabetes; targeting the program at individuals with higher HbA_1c_ or higher BMI would yield greater improvements in both HbA_1c_ and BMI.

### Diabetes Management Programs

Traditionally, diabetes management programs that supplement usual care for individuals with diabetes have focused on enhancing support and education, improving nutrition, and increasing physical activity with a structured curriculum-based approach [4]. Such intervention programs are usually conducted in-person and have been shown to be effective in improving glycemic control. In the Look AHEAD trial [48], intensive lifestyle intervention components involved group and individual meetings to achieve and maintain weight loss through decreased caloric intake and increased physical activity. The trial achieved an HbA_1c_ reduction of 0.7% and weight loss of 8.6% over 1 year [48]. In recent years, mHealth lifestyle intervention programs have emerged, bringing convenience and accessibility to individuals with diabetes, achieving HbA_1c_ reductions of approximately 0.3% to 0.5% [18,19,49-51] and insignificant changes in weight loss [19,49,51].

The personalized mHealth program in this study combined health coach-led personalized lifestyle intervention with medical support by a specialist doctor, the use of CGM, and integrated delivery through a mobile app. The medical support allowed for medication adjustments where beneficial, for example, optimizing the timing of administration of the medication to more effectively suit the lifestyle patterns of the patient. However, it is important to note that, in this study, there were no major adjustments of medications, such as initiating patients on insulin, that could have confounded the improvements observed. This is in contrast to another feasibility study with the same mHealth components, in which it was not possible to determine if improvements were due to intensification of medical therapy or from the other components of the intervention program [52].

The mobile app and CGM allowed the real-time tracking of diet, physical activity, and glucose, for interventions that were highly personalized, context sensitive, and delivered in a timely manner. Visualization of their own data, coupled with remote monitoring and actionable insights from trusted experts through the mobile app to make sense of that data, could enable the patients to appreciate the impact of their behaviors on their own health parameters. This could have further empowered and reinforced the user to implement behaviors that improve their health on a continuous, real-time basis in between clinic visits, with a low risk of adverse events such as hypoglycemia. The integrated solution incorporating medical support, CGM, and lifestyle care delivery through a mobile app likely accounted for the intervention in this study achieving results comparable to those reported in other diabetes lifestyle intervention programs, in a far shorter period of 3 months.

Based on the responses from the survey at the end of the program, patients found this personalized mHealth program to be beneficial in improving their diabetes control. Patients also reported that they were satisfied with the personalized mHealth program. These results suggested that patients found value in the mHealth program and were also receptive to the program. As there was no glycemic threshold effect, participation in a personalized mHealth program could be recommended to most individuals with diabetes or prediabetes, with the understanding that greater clinical improvement is seen with poorer starting glycemic control.

### COVID-19 and Implications on Chronic Disease Management

This program was conducted against the backdrop of a worsening COVID-19 pandemic, which saw Singapore undergo a national-level lockdown, termed *circuit breaker*, from April 7, 2020 to June 1, 2020. This coincided with the mHealth-anchored digital coaching phase of the program. During this period, there were widespread closures, of premises such as nonessential workplaces, schools, exercise and recreational facilities, and places of worship, along with the prohibition of all social gatherings [53]. Essential services in health care, transport, cleaning, food services, and supply chains remained open, but on a reduced capacity basis. This had several implications on the health and diabetes control of the patients.

With the closure of sports facilities, many of the patients who performed their physical activity in these locations were unable to continue doing so. Closure of workplaces and recreational facilities, as well as banning of social gatherings, meant that patients left their homes less and commuted less, resulting in lower physical activity levels [54]. The stress of being under lockdown may also have increased the consumption of ultra-processed food, which can be detrimental to diabetes control. While it is too early to assess the impact of the lockdown on diabetes control, experts anticipate a negative impact on weight as well as glycemic control [55,56].

During the circuit breaker period, the patients also had greater difficulty accessing health care. Many of the patients had their regular reviews with their primary care physicians postponed. Some of the patients in critical operational roles were confined to military camps, with some reporting difficulties getting refills of certain medications from their external health care providers, and in some instances, difficulties in communicating via the mobile app due to certain camp security restrictions. These health care access issues would have negatively impacted the patients’ diabetes care and control during this period.

The patients in this study experienced clinically significant improvements in their glycemic control (*P*=.004), weight (*P*<.001), and BMI (*P*<.001). This was in spite of the anticipated worsening of weight and glycemic control due to decreased physical activity, poorer diet, and lack of access to health care due to a national-level lockdown [54-56]. This highlights the role that a diabetes solution with an mHealth component can play in improving the management of chronic diseases, such as type 2 diabetes, especially during periods where there are barriers to accessing health care in person.

### Strengths and Limitations

This study evaluated a real-world context-sensitive mHealth-anchored intervention program with free-living patients. The program also coincidentally began during the start of the COVID-19 pandemic, with the bulk of the encounters occurring during the national-level lockdown in Singapore. This allowed for a timely study of the use of mHealth for chronic disease management just as the world needed to move toward embracing more digital solutions to limit in-person interactions.

A limitation of this study was that the program was conducted only with military personnel, which could have been expanded to include other professions so that the results could be more generalizable. However, the focus on military personnel could inform specific occupational policy changes to improve chronic disease prevention and management for active military personnel [57] and could reduce productivity lost among active personnel [58].

Another limitation was that the study consisted of a single intervention arm with no control group. Without a control group, there is a possibility that patients not undergoing the same program might still experience the same improvements with usual care during the same time period. However, this study demonstrated the feasibility of the program and also provided pilot data that can pave the way for future studies. Further explorations could be done on the improvements of the personalized mHealth program intervention and its various components.

Close to one-third of the patients had dropped out of the program. However, this was not higher than expected for a 3-month program, and we did not observe any systemic differences between the patients who completed the program compared to those who dropped out analyses (age category: *P*=.23; gender: *P*=.21; ethnicity: *P*>.99; diabetes status category: *P*=.52, medication adjustment category: *P*=.65; HbA_1c_ category: *P*=.69; BMI: *P*>.99). In spite of the small sample size, there was sufficient power for differences to be detected in the main analyses. However, there was insufficient power for a few of the subgroup analyses (beta errors were found to be >.2).

The patients were also only followed-up for 3 months until the end of the program; therefore, long-term effects of the program are unknown. This is a limitation commonly found in the review of other mHealth interventions [18,49,51], but there have been some promising indications of positive long-term outcomes [59]. This warrants additional follow-up investigations in future studies to explore whether effects are sustained after the program has ended, and whether some components could be implemented periodically in a cost-effective way to maintain the improvements achieved.

### Conclusion

The personalized mHealth-anchored intervention program demonstrated feasibility and acceptability and was able to produce significant reductions in HbA_1c_ (*P*=.004) and body weight (*P*<.001) in individuals with type 2 diabetes, in addition to usual care. The results also suggested that a program with a strong mHealth component could overcome challenges posed by COVID-19 to chronic disease management, including disruptions to in-person health care access. Further investigation is warranted to test the persistence of the results and the use of such digital therapeutics as a scalable solution to address the burden of diabetes.

## Abbreviations

BMI: body mass index
CGM: continuous glucose monitoring
HbA_1c_: glycated hemoglobin A_1c_
mHealth: mobile health
